# Introducing the IBJU patient corner new section

**DOI:** 10.1590/S1677-5538.IBJU.2023.04.03

**Published:** 2023-08-07

**Affiliations:** 1 ANDROFERT, Clínica de Andrologia e Reprodução Humana Campinas SP Brasil ANDROFERT, Clínica de Andrologia e Reprodução Humana, Campinas, SP, Brasil

We are excited to announce the launch of a new section in the International Brazilian Journal of Urology (IBJU): the Patient Corner. This new section will feature short articles, written in layperson’s terms, on specific urological diseases, procedures, or conditions to serve the patient community (1).

The inspiration for the Patient Corner comes from the successful ‘Patient Page’ section of the Journal of the American Medical Association (JAMA). Our goal is to expand the mission of IBJU, which is to publish high-quality urological research, by adding a service to the patient community as well.

We invite authors interested in submitting articles to the Patient Corner to write to the Editor-in-Chief to obtain approval to develop the proposed theme. All Patient Corner articles will be peer-reviewed by members of the Editorial Board and Editors and will receive a DOI. The submissions will be fully indexed in PubMed.

As a general guideline, Patient Corner articles should cover the following aspects: the problem(s) associated with the condition under discussion, how common the disease/condition is and the associated risk factors, the signs and symptoms, how the condition is diagnosed, and the treatments available. All articles should include a high-quality graphic abstract summarizing the main aspects discussed in the text. The article title should be concise and formulated as a short question.

In this issue of IBJU, we feature the first article of this new section, titled ‘What is Varicocele?’ We hope this article and the subsequent Patient Corner articles will provide patients with easy-to-understand information about their urological health.

It is essential to note that the information and recommendations featured in the Patient Corner are appropriate in most instances, but they are not a substitute for medical diagnosis. For specific information concerning the featured medical conditions, IBJU recommends patients consult a urologist.

We proudly offer the IBJU Patient Corner as a public service to the patient community. The Patient Corner articles will be freely available for download and photocopying non-commercially by physicians and other healthcare professionals to share with patients.

We hope that the Patient Corner will provide a valuable resource for patients seeking information about their urological health, and we look forward to receiving your submissions.

Sandro C. Esteves,

Associate Editor
